# Diarrheal Diseases in Under-Five Children and Associated Factors among Farta District Rural Community, Amhara Regional State, North Central Ethiopia: A Comparative Cross-Sectional Study

**DOI:** 10.1155/2020/6027079

**Published:** 2020-04-22

**Authors:** Yilkal Tafere, Bedilu Abebe Abate, Habtamu Demelash Enyew, Amsalu Belete Mekonnen

**Affiliations:** ^1^Department of Public Health, College of Health Sciences, Debre Markos University, Debre Markos, Ethiopia; ^2^Department of Public Health, College of Health Sciences, Debre Tabor University, Debre Tabor, Ethiopia; ^3^Department of Nursing, College of Health Sciences, Debre Tabor University, Debre Tabor, Ethiopia

## Abstract

**Background:**

Diarrheal diseases are the major cause of morbidity and mortality among under-five children in low- and middle-income countries including Ethiopia. One of the national initiatives to reduce its burden is an implementation of an open-defecation-free program. However, information related to the comparison of diarrheal diseases among residents in open-defecation-free and non-open-defecation-free. Hence, this study assessed the magnitude of diarrheal diseases among residents in open-defecation-free and non-open-defecation-free areas of Farta District, North Central Ethiopia.

**Methods:**

A community-based comparative cross-sectional study was conducted among 758 households (378 in open-defecation-free and 380 in non-open-defecation-free kebeles) who have under-five children using a structured questionnaire. A systematic sampling technique was used to select study participants. Binary logistic regression was used to analyze factors associated with diarrheal diseases in the district.

**Results:**

Overall, 29.9% of children had diarrheal diseases in the last two weeks prior to the study. The magnitude of diarrheal diseases among under-five children living in open-defecation-free and non-open-defecation-free residents was 19.3% and 40.5%, respectively. Lack of functional handwashing facilities (AOR: 11, 95% CI (8.1–29.6)), improper excreta disposal (AOR: 3.84, 95% CI (2.15–5.65)), and residing in non-open-defecation-free areas (AOR: 2.4, 95% CI (1.72–3.23)) were factors associated with diarrheal diseases.

**Conclusions:**

The prevalence of diarrhea among children residing in open-defecation-free areas was lower than that among children those who resided in non-open-defecation-free areas. Lack of functional handwashing facilities, residing in non-open-defecation-free areas, and improper excreta disposal were significantly associated with diarrheal diseases in the district. Strengthening health promotion on non-open defecation, maintaining functional handwashing facilities, and preparing additional handwashing facilities are necessary. Continuous engagement of the community health extension workers is recommended, sustaining the implementation of open-defecation-free programs in the district.

## 1. Background

Diarrhea refers to either an increased stool frequency or decreased stool consistency. It is defined as three or more loose or watery stools within 24 hours [1, 2]. Diarrhea is aggravated by a lack of access to sanitary disposal of waste [3]. Diarrheal diseases are a public health problem in Ethiopia [4]. According to EDHS 2016, 12% of children had diarrhea [5]. Diarrheal diseases are associated with poor environmental conditions. Ethiopia is one of the countries in which open defecation (OD) is still practiced. Most of the populations in rural areas defecate in an open field [6–8].

Similarly, in Ethiopia, graduating model families for the health extension program (HEP) and implementing community-led total sanitation and hygiene (CLTSH) are the strategies to address diarrheal diseases and other environment-related health problems in Ethiopia. The prevalence of diarrhea among children whose families were nonmodel for health extension programs ranged from 14% in Hawassa to 25.5% in Sheko [9, 10]. However, the magnitude was lower among model families and ranged from 6.4% to 9% [9, 10]. The study done in Kersa District, Jimma Zone, Ethiopia, revealed that the prevalence of diarrheal diseases among children living in CLTSH-implemented and nonimplemented areas was 18.9% and 22.2%, respectively [11]. Diarrheal diseases in children ranged from 6.5% in Hulet Ejju Enessie in North Western Ethiopia to 33.2% in Assosa, Western Ethiopia [12–16].

Studies done in Ethiopia in various corners of the country had reported that lack of latrine, faces seen around the house, lower maternal education, lower socioeconomic status, poor handwashing practice, improper refuse disposal, improper children's faeces disposal, and being nonmodel households were associated with children to develop diarrheal diseases [9–11, 13, 16–18].

To avert diarrheal diseases, Ethiopia has adopted the CLTSH strategy. This strategy is part of the health extension program and is implemented by health extension workers. Community-led total sanitation targets on ending open defecation, hygienic use of toilets, and handwashing at critical times. Little is known about the effect of the ODF on diarrheal diseases in children in Ethiopia in general and in the study area in particular. Therefore, this study tried to compare the magnitude of diarrheal diseases among children who resided in ODF and NODF kebeles in Farta District, in Northwestern Ethiopia.

## 2. Methods

### 2.1. Study Setting and Study Period

The study was conducted in Farta District. Farta District is one of the districts of South Gondar Zone, North Central Ethiopia. It is located 105 kilometres from Bahir Dar (the administrative town of the Amhara Region) and 666 kilometres far away from Addis Ababa, the capital city of Ethiopia. The district has a total of 43 kebeles (*Kebeles are the smallest administrative unit in Ethiopia*) (41 rural and 2 towns). Among which 31 kebeles are ODF areas and the rest 12 kebeles are non-open-defecation-free areas. All mothers who had under-five children were the source population, whereas all mothers who had under-five children in selected ODF and NODF kebeles were the study population. The study was conducted in Farta District from January 1 to 30, 2017.

### 2.2. Study Design

This is a comparative cross-sectional study.

### 2.3. Sample Size and Sampling Techniques

The sample size was determined using the STATCALC program of EPI INFO statistical software version 7 by considering 18.91% prevalence of diarrheal disease among households living in ODF and 22.22% in NODF areas [15], 95% confidence interval, 80% power, and open defecation free ODF and non-defecation free households ratio 1 : 1, to detect 2 odds ratio, design effect 2. The final total sample size of 772 (386 ODF and 386 NODF) HHs with under-five children was included in the study. The study subjects were selected by the stratified sampling procedure. HHs were stratified as ODF and NODF areas. Then, 5 kebeles were selected from each stratum. Three hundred eighty-six households were selected from both ODF and NODF residents. Proportional allocation was employed for kebeles. Finally, a systematic sampling technique was used to select each eligible HH. Mothers in these households were finally recruited and interviewed.

### 2.4. Data Collection Procedures

Data were collected through face-to-face interviews using a structured questionnaire depending on different literatures [9, 10]. Ten trained female nurses who have diploma and ample experiences had collected the data.

### 2.5. Data Quality Assurance Procedures

Data quality was ensured before, during, and after data collection. The tool was developed after thorough review of related literatures. Two-day training was given. The questionnaire was pretested to check sequence of questions, language, comprehension, and understanding of questions by respondents among the participants. It was conducted in 5% of the sample size around Addis Zemen Town which is out of the main study area. Findings of pretesting were discussed among data collectors and researchers so that the tool was modified accordingly.

Supervision and follow-up of data collectors was conducted including observation of how they are administering questions and approaching the respondents. The filled questionnaires were checked for completeness by data collectors and the researchers on a daily basis. Consequently, any problems encountered were discussed among the survey team and solved immediately.

The questionnaire was pretested to check sequence of question, language, comprehension of the questions among the participants, and duration of an interview on five percent of the sample size among health workers of Addis Zemen health facilities which was excluded out of the main study. The final interview was conducted using the modified questionnaire. Every problem during data collection was solved through contact with supervisors. Data cleaning was done before analysis.

### 2.6. Data Analysis

After checking for completeness of data, they were coded and entered into Epi-Info version 7 databases and then exported to SPSS for analysis. Crude and adjusted odds ratio with 95% CI was calculated. A *P* value less than 0.05 was considered as a level of significance. Bivariate and multiple logistic regressions were done.

### 2.7. Ethical Approval

Ethical clearance was obtained from the Debre Tabor University Research Ethics Review Committee. A permission letter was obtained from the Farta District Health Office. The participants were informed that participation is made entirely voluntarily, and written consent was taken from the mothers/caretakers of the under-five children before the actual data collection. For the affected children at the time of data collection, the respondent was advised to get medical treatment to the nearest health institution.

## 3. Results

### 3.1. Sociodemographic Characteristics of Study Participants

A total of 758 mothers with under-five children (378 open-defecation-free and 380 non-open-defecation-free kebele residents) participated in the study making a response rate of 98.19%. Out of these, 736 (97.1%) of respondents were biological mothers of the under-five children. The mean age of the respondents was 31.66 ± 6.74 standard deviations (SD) years, and 180 (23.7%) of the respondents were between the age group of 25 and 29 years. Nearly all mothers 736 (97.1) were married, and the majority of them 692 (91.3%) were housewives.

Regarding the educational status of the study participants, 420 (55.4%) of them had no formal education. The mean family size of the respondent was 5.1 ± 1.53 SD. About six in ten, 474 (62.5%), of households had a family size of less than five of which 221 (29.2%) HHs were in ODF and 253 (33.4%) were in non-open-defecation-free (NODF) kebeles. Half, 378 (49.92%), of the HHs had family income between 1001 and 2000 Ethiopian Birr per month (Table 1).

### 3.2. Household and Environmental-Related Characteristics of Study Participants

More than five in ten (45.6%) of residential houses had less than three rooms. About 322 (85.2%) households in open-defecation-free kebeles and 56 (14.8%) households in non-open-defecation-free kebeles had separate kitchens, respectively. Nearly nine in ten, 328 (87.7%), HHs of open-defecation-free kebeles and 46 (12.3%) HHs in non-open-defecation-free kebeles had a separate room for animals correspondingly. About two-thirds, 507 (66.9%), of households had latrine, out of which 340 (89.9%) were in open-defecation-free kebeles. Nevertheless, less than half, 167 (43.9%), of the HHs in non-open-defecation-free (NODF) kebeles had latrine. Most, 492 (97%), of the latrines were pit latrines. Of these latrines, 83 (24.4%) were in ODF kebeles. About one-third, 61 (36.5%), of NODF households' latrine was constructed 4 or more years prior to the study. Among households who had a latrine, 340 were in open-defecation-free kebeles and all (100%) of the latrines were functional. Among households who had latrine, 159 were in non-open-defecation-free kebeles and 95.2% latrines were functional. One hundred sixty-five (47.7%) HHs residing in open-defecation-free kebeles and 88 (38.6%) HHs in non-open-defecation-free kebeles who had latrine also had handwashing facilities (Table 2).

### 3.3. Demographic Characteristics of Under-Five Children of Study Participants (Mothers or Caregivers)

The mean age of under-five children was 21.3 months ±13.9 months (SD). About half of them, 401 (52.9%), were male, whereas females were 357 (47.1%). The majority of the children (77.0%) were born in the health facility, and the rest were born at home. Third quartiles of the children (76.4%) were fully immunized, and the rest (5.4%) were not immunized.

### 3.4. Prevalence of Diarrheal Disease among Under-Five Children

The overall prevalence of diarrheal diseases among under-five children was 29.9% of which 73 (19.3%) were from children who dwell in ODF kebeles and 154 (40.5%) were from children who dwell in NODF kebeles. Diarrheal diseases were most prevalent among children of aged 24–59 months (100 (13.2%)). About 17.2% of children's mothers had the diarrheal diseases.

### 3.5. Factors Associated with Diarrheal Diseases among Under-Five Children

In binary logistic regression, not attending formal education (AOR: 2.1, 95% CI (1.2, 2.7)), family size ≥5 (AOR: 1.3, 95% CI (1.11, 1.9)), low monthly income of HHs (AOR: 2.1, 95% CI (1.3, 2.)), age of the child >12 months (AOR: 2, 95% CI (1.4, 2.7)), owning latrines for ≥4 years, living in NODF hebeles (AOR: 2.5, 95% CI (1.17, 3.23)), and compounds in which faeces were seen around (AOR: 3.84, 95% CI: (2.15, 5.65)) were significantly associated with diarrheal diseases.

In multivariable logistic regression, having nonfunctional handwashing facilities and residing with families who did not wash their hands were significantly associated with under-five diarrheal diseases. Diarrheal disease had occurred 11 times more likely (AOR: 11, 95% CI (8.1, 29.6)) to occur among children in HHs who have no functional handwashing facilities. Children who lived with HHs whose families did not wash their hands after touching infants' faeces were 1.6 times more likely to develop diarrheal diseases (AOR: 1.6, 95% CI (1.12, 2.03)) (Table 3).

## 4. Discussion

In this study, the overall two weeks prevalence of diarrheal diseases was 29.9%. This is similar to a study done in Nekemte Town (28.9%) [14] and higher than studies conducted in Hulet Ejju Enessie (6.5%), Mecha (18%), and EDHS 2016 (12%), respectively [5, 15, 16]. However, it is slightly lower than the studies done in Assosa Town which reported 33.2% and the Debre Birhan Town that reported 31.7% [12, 13].

Similarly, in this study, the prevalence of diarrheal diseases among children residing in NODF kebeles was 40.5%. It is about 20% higher than that of those under-five children who resided in ODF kebeles which was 19.3%. This finding was supported by the study done in Kersa that CLTSH implementation reduces diarrheal diseases among children living in HHs compared to nonimplemented kebeles (18.91% and 22.22%, respectively) [11]. This difference might be due to the fact that proper disposal of the excreta implementation strategy of the government; capacity building of families to be role model to waste management, strict follow-up by health extension workers to build the capacity of the community and, support to those HHs living in ODF kebeles. This support may bring knowledge and skill development and access for excreta disposal infrastructure and community involvement in waste management issues to those HHs living in open-defecation-free kebeles, and it made them practice proper excreta disposal compared to those HHs living in non-open-defecation-free kebeles.

Seven in ten (71.3%) of the respondents disposed faeces around the house either in the bush or outside the compound, while only 28.7% of them had disposed in the latrine. The use of safe disposal of children's faeces in the present study was lower when compared with the study in Hulet Ejju Enessie; the prevalence of disposing faeces in the latrine was 65.9% [15]. Barriers to the transmission of enteric pathogens are safe excreta disposal. Open-field disposal did not remove the pathogen, rather the pathogenic organism reserves in the soil and later through the feco-oral route; it was reingested, resulting in repeated episodes of diarrheal diseases.

This study found that 10% of HHs residing in ODF and 251 (56.1%) HHs living in NODF kebeles had no latrine. Faeces were seen in the compound of about one in three of them (67.3% HHs) living in ODF and non-open-defecation-free (NODF) kebeles, respectively. From children who had developed diarrheal diseases, faeces were seen in nine in ten of their compounds. The difference may be due to the expansion of the HEP and the implementation of the open-defecation-free (ODF) strategy at a community level. This shows the possible effect of the ODF implementation strategy towards reducing diarrheal diseases by half among children; hence, these programs are noted to play a great role in the prevention of diarrheal diseases. The availability of latrine reduces contamination of the environment by excreta, and this further prevents transmission of disease-causing organisms [6]. This study found that 10% HHs living in ODF kebeles returned to NODF kebeles.

Not attending formal education, low monthly income, and family size >5 were significantly associated with diarrheal diseases. This finding is supported by studies [9, 10, 13, 16]. Socioeconomic status and level of education might have a direct effect on environmental hygiene practices. Children who live in NODF kebeles were 2.4 times more likely to develop diarrheal diseases than those who live in ODF kebeles. The possible reason might be that when there is improper disposal of waste, flies might have access for faeces, and it can contaminate the soil, food, and water. These, combined with other situations, further predispose children to diarrheal diseases. This difference might be indicated by the possible effect of the ODF strategy among HHs. During the implementation of the ODF strategy at community level follow-up of health professionals, local administrators might be attributed to the behaviour changes being observed.

The diarrheal diseases were nearly two times more likely (AOR: 1.8, 95% CI (1.2, 3.1)) among children whose families owning latrines for ≥4 years than owning latrines for less than four years of service. The disposal method of child faeces was found to be statistically significant with diarrheal diseases. Children from those HHs in which child faeces were throwing out of houses had about 1.4 times more likely to have diarrhea compared to those children from houses in which child faeces had disposed in the latrine (AOR: 1.4, 95% CI (1.1, 3.7)). This finding was supported by the studies conducted in Hawassa and Nekemte [10, 14].

In this study, children from those HHs in which faeces were observed around the compound had 28 times more likely to have developed diarrhea compared to those children from houses in which faeces had not been observed around the compound (AOR: 27.6, 95% CI (18.9, 37)). This finding is consistent with studies done in Sheko, Nekemte, Hawassa, and Cameroon [9, 10, 14, 18]. When children live in an unhygienic compound, they will be more likely to have poor personal hygiene that leads to diarrheal diseases. Handwashing after touching the infant's faeces was found statistically significant with diarrheal diseases. This finding is consistent with studies done in Kabul, Afghanistan, that washing and the presence of improved sanitation facilities were protective for diarrheal diseases [17]. However, this study does have some inherent limitations. The prevalence of diarrheal may not reflect the actual situation that may be observed in the various seasons of the year.

## 5. Conclusions and Recommendations

Despite encouraging gains made in the reduction of diarrheal diseases among children living in ODF kebeles, this study demonstrated that the overall prevalence of diarrheal diseases in under-five children remains high in the study area. Significant numbers of respondents in ODF and NODF kebeles had no latrine. Low household income, not attending formal education, improper excreta disposal, living in non-open-defecation-free kebeles, and lack of handwashing facilities were significantly associated with occurrence of diarrheal diseases among under-five children. To sustain and further strengthen the achievements gained, communities should accelerate their concerted efforts to open-defecation-free implementation.

## Figures and Tables

**Table 1:** Sociodemographic characteristics of mothers/caregivers of under-five children in Farta District, North Central Ethiopia, 2017 (*n* = 758).

Variables	Status of kebeles				Total	
	ODF dwellers		NODF dwellers			
*Age of mothers/caregivers*	No.	%	No.	%	No.	%
15–19	0	0	8	2.1	8	1.1
20–24	60	15.9	67	17.6	127	16.8
25–29	86	22.8	94	24.7	180	23.7
30–34	88	23.3	80	21.1	168	22.2
35–39	85	22.5	73	19.2	158	20.8
40–44	44	11.6	46	12.1	90	11.9
45–49	15	4.0	12	3.2	27	3.6

*Educational status*						
Cannot read and write	227	29.9	193	25.5	420	55.4
Read and write	98	12.9	70	9.2	168	22.2
Primary	42	5.5	87	11.5	129	17
Secondary	11	1.5	30	4.0	41	5.4

*Occupation of the respondent*						
Housewife	384	50.7	308	40.6	692	91.3
Farmer	30	4	36	4.7	66	8.7
*Size of the households*						
<5	221	29.2	253	33.4	474	62.5
≥5	157	20.7	127	16.8	284	37.5

*Family average monthly income*						
≤1000	38	5.0	112	14.0	150	19.8
1001–2000	202	26.6	176	23.2	378	49.9
>2000	138	18.2	92	12.1	230	30.3

**Table 2:** Household and environmental-related characteristics of study participants among HHs in open-defecation-free kebeles and non-open-defecation-free kebeles in Farta District, North Central Ethiopia, February, 2017 (*n* = 758).

	ODF HHs (378)	NODF HHs (380)
Variables	*N* (%)	*N* (%)
*Type of roof of the house*		
Corrugated iron	317 (83.9)	316 (83.2)
Thatched roof	61 (16.1)	64 (16.8)

*Type of floor of the house*		
Earthen floor	375 (99.2)	380 (100)
Cemented	3 (0.8)	0 (0.0)

*Type of wall of the residential house*		
Mud	371 (98.1)	380 (100)
Cemented	7 (1.9)	0 (0)

*Presence of separate kitchens*		
Yes	322 (85.2)	316 (83.2)
No	56 (*14*.8)	64 (16.8)

*Presence of separate rooms for domestic animals*		
Yes	328 (87.7)	350 (92.1)
No	46 (12.3)	30 (7.9)

*Number of rooms in the houses*		
<3	143 (37.8)	203 (53.4)
≥3	235 (62.2)	177 (46.6)

*Availability of latrine*		
Yes	340 (89.9)	167 (43.9)
No	38 (10.1)	251 (56.1)

*Service year of latrine*		
≤4 years	257 (75.6)	106 (63.5)
>4 years	83 (24.4)	61 (36.5)

*The pattern of latrine use*		
Always	273 (80.3)	63 (37.)
Most of the time	48 (14.1)	29 (17.4)
Sometimes	19 (5.6)	75 (44.9)

*Place of infants' excreta disposal*		
Throwing faeces out of houses	252 (66.7)	288 (75.8)
Dispose in the latrine	126 (33.3)	92 (24.2)

*Handwashing practice after touching infants' faeces*		
Yes	150 (39.7)	217 (57.1)
No	228 (60.3)	163 (42.9)

**Table 3:** Factors associated with diarrheal diseases among children under five years of age living in ODF and NODF kebeles in Farta District, North Central Ethiopia, 2017.

Variables	Diarrhea			AOR (95%, CI)	*P* value
	Yes (%)	No (%)	COR (95% CI)		
*Educational status of respondents*					
Have no formal education	150 (66.1)	438 (82.5)	2.41 (1.6, 3.5)	2.1 (1.2, 2.7)	*P* < 0.01
Have formal education	77 (33.9)	93 (17.5)		1.00	
*Family average monthly income*					
≤1000	36 (15.9)	114 (21.5)	2.39 (1.52, 3.78)	2.1 (1.3, 2.9)	*P* < 0.008
1001–2000	92 (40.5)	268 (53.9)	2.35 (1.65, 3.35)	1.2 (1.1, 2.2)	*P* < 0.001
>2000	99 (43.6)	131 (24.7)	1.00	1.00	
*Family size*					
<5	126 (55.5)	348 (65.5)	1.00	1.00	
≥5	101 (44.5)	183 (34.5)	1.5 (1.2, 2.10)	1.3 (1.11, 1.9)	*P* < 0.02
*Age of the child*					
≤12 months	50 (22)	196 (36.9)	1.00	1.00	
>12 months	177 (78)	335 (63.1)	2.07 (1.45, 2.97)	2 (1.4, 2.7)	*P* < 0.001
*Number of rooms in the house*					
1-2 rooms	90 (39.6)	256 (48.2)	1.42 (1.3, 1.94)	1.3 (1.1, 1.7)	*P* < 0.03
3 and more rooms	137 (60.4)	275 (51.8)	1.00	1.00	
*Type of house roof constructed*					
Grass	18 (7.9)	107 (20.2)	2.9 (1.73, 4.96)	2.3 (1.53, 3.4)	*P* < 0.01
Corrugated iron sheet	209 (92.1)	424 (79.8)	1.00	1.00	
*Status of kebeles*					
NODF	154 (67.8)	226 (42.6)	2.8 (2.05, 3.95)	2.4 (1.17, 3.23)	*P* < 0.03
ODF	73 (32.2)	305 (57.4)	1.00	1.00	
*Latrine utilization pattern*					
Sometimes	44 (29.3)	40 (11)	1.6 (1.30, 2.27)	1.50 (1.1, 2.4)	*P* < 0.001
Most of the time	55 (36.7)	59 (16.3)	4.82 (2.1, 6.93)	2.6 (1.9, 3.7)	*P* < 0.002
Always	51 (34.0)	264 (72.7)	1.00	1.00	
*Service year of latrine*					
0–4 years	90 (60)	273 (76.5)	1	1	
>4 years	60 (40)	84 (23.5)	2 (1.44, 3.26)	1.8 (1.2, 3.1)	*P* < 0.001
*Functional handwashing facility near the latrine*					
Yes	144 (26.4)	129 (70.2)	1	1	
No	15 (73.6)	61 (29.8)	11.9 (6.2, 23.1)	11 (8.1, 29.6)	*P* < 0.001
*Where do you dispose infants' excreta?*					
Throwing out of houses	176 (77.5)	364 (68.5)	1.6 (1.2, 3.9)	1.4 (1.1, 3.7)	*P* < 0.001
Dispose in the latrine	51 (22.5)	167 (31.5)	1.	1	
*Mothers had a diarrheal diseases in the last 2 weeks*					
Yes	49 (21.6)	81 (15.3)	11.5 (1.03, 2.27)	1.2 (1.1, 24)	*P* < 0.006
No	178 (78.4)	450 (84.7)	1.00	1.00	
*Handwashing after touching infants' faeces*					
Yes	137 (60.4)	230 (43.3)	1.00	1.00	
No	90 (39.9)	301 (56.7)	2.8 (1.45, 3.73)	1.6 (1.12, 2.03)	*P* < 0.001
*Faeces seen around the compound*					
Yes	181 (92.8)	111 (29.9)	30.3 (16.8, 54)	27.6 (18.9, 37)	*P* < 0.004
No	14 (7.2)	260 (70.1)	1.00	1.00	

## Data Availability

The data used to support the findings of this study are available from the corresponding author upon reasonable request.

## Abbreviations

AOR: Adjusted odds ratio
CI: Confidence interval
CLTSH: Community-led total sanitation and hygiene
DD: Diarrheal diseases
EDHS: Ethiopian Demographic Health Survey
EPI INFO: Epidemiological information
HEP: Health Extension Program
HHs: Households
NODF: Non-open-defecation-free
ODF: Open-defecation-free
OR: Odds ratio
SPSS: Statistical Package for the Social Sciences
WHO: World Health Organization.
